# *In Silico* Study Reveals How E64 Approaches, Binds to, and Inhibits Falcipain-2 of *Plasmodium falciparum* that Causes Malaria in Humans

**DOI:** 10.1038/s41598-018-34622-1

**Published:** 2018-11-06

**Authors:** Emmanuel Oluwatobi Salawu

**Affiliations:** 1 Insilico Taiwan, Taipei City, Taiwan; 2 TIGP Bioinformatics Program, Academia Sinica, Taipei City, Taiwan; 3 Institute of Bioinformatics and Structural Biology, National Tsing Hua University, Hsinchu City, Taiwan; 4 School of Computer Science, University of Hertfordshire, Hatfield, United Kingdom; 5 Bioinformatics Center, Sheridan, WY USA

## Abstract

*Plasmodium falciparum* malaria, which degrades haemoglobin through falcipain-2 (FP2), is a serious disease killing 445 thousand people annually. Since the *P*. *falciparum’s* survival in humans depends on its ability to degrade human’s haemoglobin, stoppage or hindrance of FP2 has antimalarial effects. Therefore, we studied the atomic details of how E64 approaches, binds to, and inhibits FP2. We found that E64 (1) gradually approaches FP2 by first interacting with FP2’s D170 and Q171 or N81, N77, and K76; (2) binds FP2 tightly (ΔG_binding_ = −12.2 ± 1.1 kJ/mol); and (3) persistently blocks access to FP2’s catalytic residues regardless of whether or not E64 has already been able to form a covalent bond with FP2’s C42. Furthermore, the results suggest that S41, D234, D170, N38, N173, and L172 (which are located in or near the FP2’s catalytic site’s binding pocket) contribute the most towards the favourable binding of E64 to FP2. Their *in silico* mutations adversely affect E64-FP2 binding affinity with D234L/A, N173L/A, W43F/A, D234L/A, H174F/A, and N38L/A having the most significant adverse effects on E64-FP2 binding and interactions. The findings presented in this article, which has antimalarial implications, suggest that hydrogen bonding and electrostatic interactions play important roles in E64-FP2 binding, and that a potential FP2-blocking E64-based/E64-like antimalarial drug should be capable of being both hydrogen-bond donor and acceptor, and/or have the ability to favourably interact with polar amino acids (such as S41, S149, N38, N173, N77, Q171) and with charged amino acids (such as D234, D170, H174) of FP2. The abilities to favourably interact with ASN, ASP, and SER appears to be important characteristics that such potential drug should have.

## Introduction

Malaria is one of the most important public health and clinical health problems in many tropical (especially Tropical-African) countries killing about 445 thousand people annually^1^ of which more than 50% are children under the age of 5 years^1–3^. Although malaria has been known to humans since 2700 BC^4^ and malaria parasites were first isolated in Constantine, Algeria, on the 6th of November 1880 (by Charles Louis Alphonse Laveran, a French army surgeon, who later won the Physiology or Medicine Nobel Prize in 1907 for this discovery)^4–6^, the malaria problem remains unsolved. Despite the efforts targeted at combating malaria disease^7–10^, malaria is neither eradicated nor eliminated at the moment^11–13^, and malaria epidemic in the tropical-developing nations appears to be largely neglected^14–16^ by the developed nations that have more resources at their disposal.

*P*. *falciparum* (as well as *P*. *vivax*, *P*. *ovale*, *P*. *knowlesi*, and *P*. *malariae* to a much smaller extent) causes malaria^17^. To survive in its human host, *P*. *falciparum* (which is the most virulent plasmodium species) must use its cysteine proteases, namely falcipain-1 (FP1), FP2, and FP3 to degrade human haemoglobin^18^. In other words, the survival of *P*. *falciparum* in humans depends on its ability to degrade humans’ haemoglobin. Indeed, any stoppage or even hindrance of the ability of *P*. *falciparum* to degrade the human haemoglobin through its cysteine protease kills *P*. *falciparum*^19^ most especially at the Trophozoite stage of its development, when the FP2 is of great importance to the *P*. *falciparum*^20^. This makes FP2 an important antimalarial drug target because its inhibition kills the malaria-causing *P*. *falciparum* (at least at its trophozoite stage).

**Figure 1:**
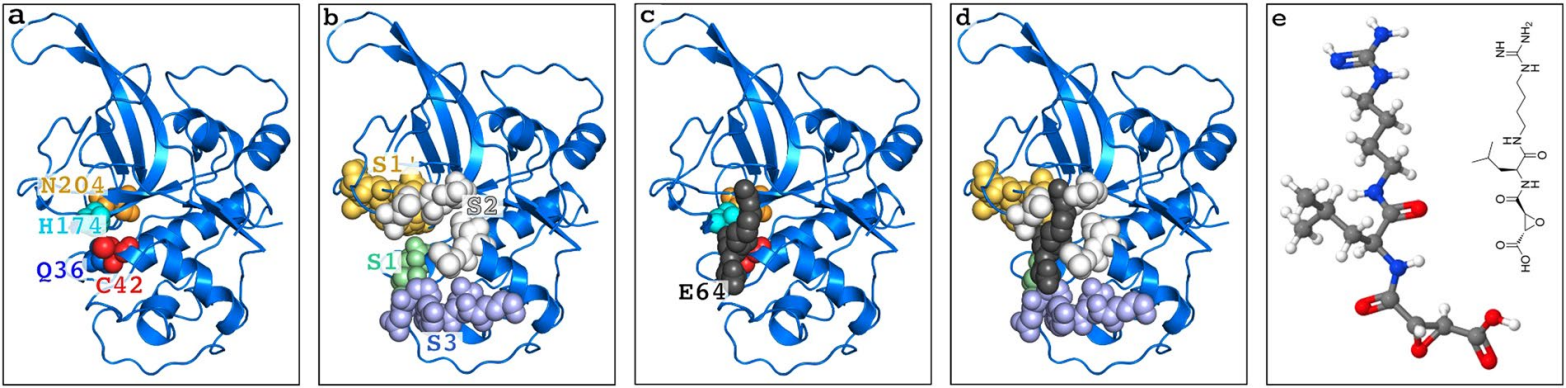
The structure of FP2-E64 complex based on 3BPF in the PDB and the Chemical Structure of E64. The positions of the catalytic residues, Q36, C42, H174, and N204 (Q36 is located behind C42 in the current view), are shown as spheres in (**a**). The known binding pocket subsites, S1 (green), S2 (grey), S3 (pale blue), and S1’ (orange) are shown as spheres in (**b**). The position of E64 (the black spheres) relative to the catalytic residues is shown in (**c**), and relative to the binding pocket subsites is shown in (**d**). The structure of E64 is shown in (**e**).

Inhibition of FP2 (Fig. 1a,b) can be achieved with E64 (Fig. 1c–e), an epoxide that is able to inhibit various cysteine proteases such as cathepsin B, cathepsin L, papain, staphopain and calpain^21^. The strong cysteine-proteases-inhibition ability of E64^22–24^ makes it a good antimalarial drug precursor.

Although the crystal structure of FP2-E64 complex exists in the Protein Data Bank (PDB ID: 3BPF)^25^, the time-resolved dynamical mechanisms by which E64 (1) approaches FP2, (2) tightly binds to FP2 (before eventually forming a covalent bond with FP2), and (3) inhibits FP2 have never been (to the best of our knowledge) explicitly studied especially at the atomic level. In addition, the detailed molecular dynamics accompanying the approach of FP2 by E64 and the characterization of their initial encounters and binding dynamics have never been studied.

Here, we present the atomic details of how E64 approaches and binds to FP2, and the dynamic interactions between E64 and FP2 (at the early and later stages of the binding). In addition, we give accounts of the factors that promote the stability of the FP2-E64 complex and their interaction prior to and after the formation of E64-FP2 covalent bond. Furthermore, we show that E64 persistently blocks the active site of FP2 in a considerably consistent manner. We also identified the residues of FP2 that contribute the most towards the favourable binding of E64 to FP2, and show how their *in silico* mutations to alanine, phenylalanine, or leucine adversely affect E64-FP2 binding Gibbs free energy change. The information presented in this paper has the potentials of guiding the design of E64-based/E-64-like antimalarial drugs that target FP2.

## Results and Discussions

### How E64 Approaches FP2

**Figure 2:**
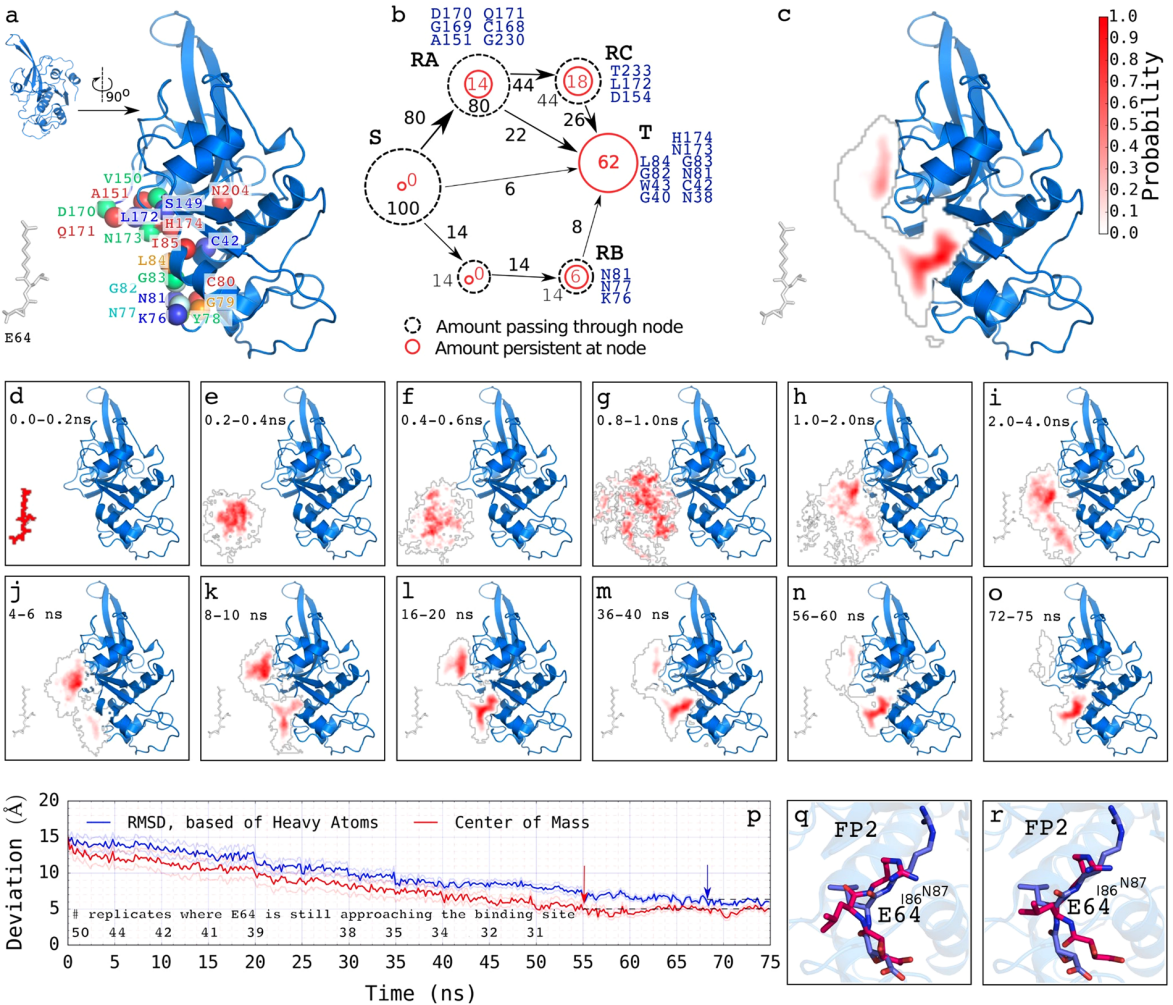
How E64 approaches and binds to FP2. The results from the 50 independent unbiased explicit-solvent MD simulations (75 ns each, 3,750 ns in all) are summarised here. (a, inset) The orientation of FP2 presented in every other figures are videos. The molecular system is rotated by about 90° counter-clockwise to obtain the orientation of the FP2 presented in this Fig. 2. This is done to enhance the visualization of how E64 approaches FP2 presented here in Fig. 2. (**a**) A typical structure used for each of the explicit-solvent MD simulations wherein E64 is placed 15 Å away from FP2. Some of the residues of FP2 found to be important are shown as coloured spheres. A model depicting (approximately) how E64 leaves the source, S, and approaches FP2 via the residues at recruiter region A (RA, then RC) and at RB, and eventually arrives at the target binding site, T, is shown in (**b**). The overall regions frequently sampled by E64 as it approaches and binds to FP2 are shown in (**c**). The regions that E64 occupies with high probability are shown in dark red (and low probability are shown in gradients of pale red to white). Regions that E64 occupies with probabilities less than 0.05 are not coloured/not highlighted. The average regions occupied by E64 relative to the position of FP2 across the trajectories from 0.0 ns to 75.0 ns are shown in (**d**–**o**). The probabilities depicted by the shades of red in each panel are based on the segment of the trajectory that the panel represents. For example, the occupancy probability shown in panel (**i**) is computed from the frames/snapshots that made up 2.0 ns to 4.0 ns segment of the trajectory. A quantitative representation of how E64 approaches FP2, whereby the average deviations of its structures and positions from the X-ray crystallography structure and position shows overall decrease with time for the 31 trajectories where the target, T, was reached (i.e. centre of mass within 5 Å) are shown in (**p**). Although the curves are based on the trajectories where the target, T, was reached, the number of trajectories where E64 continued to proceed towards the target, T, are shown above the horizontal axis line in (**p**) for every 5 ns where the number changed. The red arrow pointing to the broken black line shows the point where the movement of E64 to the binding site appears to have reached an equilibrium and plateaued. The blue arrow pointing to the broken black line shows the point where the RMSD of E64 from the target appears to have reached an equilibrium and plateaued. Panels (q) and (r) show examples of E64 obtained with positions and conformations closest (**q**: RMSD of 3.66 Å; and **r**: 3.68 Å) to the E-64 derived from the X-ray structure of FP2-E64 complex. The leucine-like nitrogen-rich side chain of E64 is able to transiently form hydrogen bonds with the backbone of I86 and/or the side chain of N87 of FP2.

Our 50 independent explicit-solvent molecular dynamics (MD) simulations (75 nanoseconds each, 3,750 ns in all, initial structure shown in Fig. 2a) show that E64 mainly approaches FP2 either by first interacting with D170, Q171, C168, G169, A151, and G230 (which we call “recruiter group A”, RA, Fig. 2b) or by first interacting with K76, N77, and N81 (which we call “recruiter group B”, RB, Fig. 2b). Our results show that, most often, E64 does not directly/immediately bind to the active site of FP2 (namely H174, C42, N204, and Q36), but rather approaches FP2 by first interacting with the residues at RA and RB in about 80% and 14% of the time respectively (Fig. 2b).

Once bounded to FP2 at RA (which is made up by D170, Q171, C168, G169, A151, and G230), about 22% (out of the 80%) of the E64 bounded to FP2 at RA proceeds to finally bind to FP2 at where they block the FP2’s catalytic residues (Fig. 2b). Additional 44% (out of the 80%) of the E64 bounded to FP2 at RA proceeds to bind to FP2 at RC (which is made up by D154, L172, and T233) before they finally bind E64 near its catalytic site and block FP2’s catalytic residues (with 26% out of the 44% of E64 at RC making their way to the target, T). In a similar way, about 8% (out of the 14%) of E64 bounded to FP2 at RB proceed to the actual target, T, where they block access to the active site residues (Fig. 2b). Once bonded to FP2 at the target, T, E64 persistently blocks the active site residues of FP2 even prior to its formation of a covalent bond with the sulphur atom of cysteine 42 of FP2 (CYS42:S). The extensive details of the route through which E64 approaches and binds to FP2 are presented in Fig. 2b–p. On the average, the movement of E64 to the binding site (based on its centre of mass) appears to have reached an equilibrium and plateaued at around 55 ns (depicted by the red arrow pointing to the broken black line in Fig. 2p). While the E64 was not necessarily moving closer (based on its centre of mass) to FP2 after 55 ns, (on the average) we observed changes in its orientations and conformations that led to further decreases in its RMSD from the target (based on heavy atoms) until around 68 ns in the trajectories after which E64 did not appear to be getting closer to the target any longer (depicted by the blue arrow pointing to the broken black line in Fig. 2p). Examples of the obtained conformations of E64 having the most similar position and orientation as that seen in E64-FP2 X-ray structure are shown in Fig. 2q (RMSD: 3.66 Å) and 2r (RMSD: 3.68 Å). It may not be possible to obtain a much smaller deviation (RMSD) from the E64 in the X-ray structure as the E64 in the X-ray structure is covalently bonded to S atom of C42 of FP2, while “how E64 approaches and binds to FP2” presented here was (and can only be) studied using E64 that is not yet covalently bonded to FP2. Furthermore, the deviation between the most similar conformation observed (while studying how E64 approaches FP2) and the X-ray structure is partly due to the leucine-like nitrogen-rich side chain of E64 transiently forming hydrogen bonds with the backbone of I86 and/or the side chain of N87 of FP2. Nonetheless, the goal of studying how E64 approaches FP2 has already been realized in this aspect of this study. ABMD simulations used in a subsequent aspect of this study shows E64-FP2’s binding conformations that are very similar to that seen in the X-ray structure.

We observed that the initial interactions of E64 with FP2 (via RA and RB) are made possible (or, at least, highly facilitated) (1) by the characteristic topology of the surface of FP2 that make D170 and Q171 (at RA) or N77 and N81 (at RB), which are persistently flexible coils^26^, protrude towards E64 (Fig. 2a), and (2) by the chemical natures of the amino acids at RA and RB. Of remarkable importance are the contributions of Q171 at RA and N81 at RB, where they consistently contribute favourable electrostatic interactions, favourable van der Waals interactions, and favourable hydrogen-bonding towards the binding of E64 to FP2 (Table 1, Supplementary Table S1).

**Table 1:** Contributions of hydrogen bonds, electrostatic, and van der Waals interactions towards the favourable binding of E64 to FP2.

Amino Acids^#^	Regression Coefficients*		
	Hydrogen bonding	Electrostatic energy	van der Waals energy
N38	−49.96	0.18	0.51
C42	−11.32	0.34	
N81	−10.30	0.23	
Q171	−5.19	0.17	0.92
***D234***	−***5***.***54***	***0***.***09***	
C39	−3.64	0.27	0.69
G40	−3.27	0.40	0.64
H174	−3.06	0.76	
***G82***	−***3***.***00***	***0***.***22***	***0***.***50***
S41	−3.15	0.31	
***V152***			***2***.***19***
***K76***		***0***.***16***	***1***.***78***
***N77***			***1***.***77***
G83		0.97	0.54
F75		0.59	0.89
W43		0.26	0.95
***L172***		***0***.***38***	***0***.***75***
***Y78***		***0***.***13***	***0***.***81***
L84		0.89	
D170			0.81
Q36		0.76	
***W206***		***0***.***42***	

^*^Dependent variable is the free energy change accompanying the binding of E64 to FP2. Only the statistically significant (*p* < *0*.*05)* regression coefficients contributing favourably towards the binding of E64 to FP2 are shown. The full model, including the non-statistically significant regression coefficients, is shown in Supplementary Table S1. *The coefficient of determination (R*^*2*^*)* = *67*.*9%*. ^*#*^*Residues in the binding pocket subsites of FP2 are written in italics*.

### E64 Strongly Binds FP2

**Figure 3:**
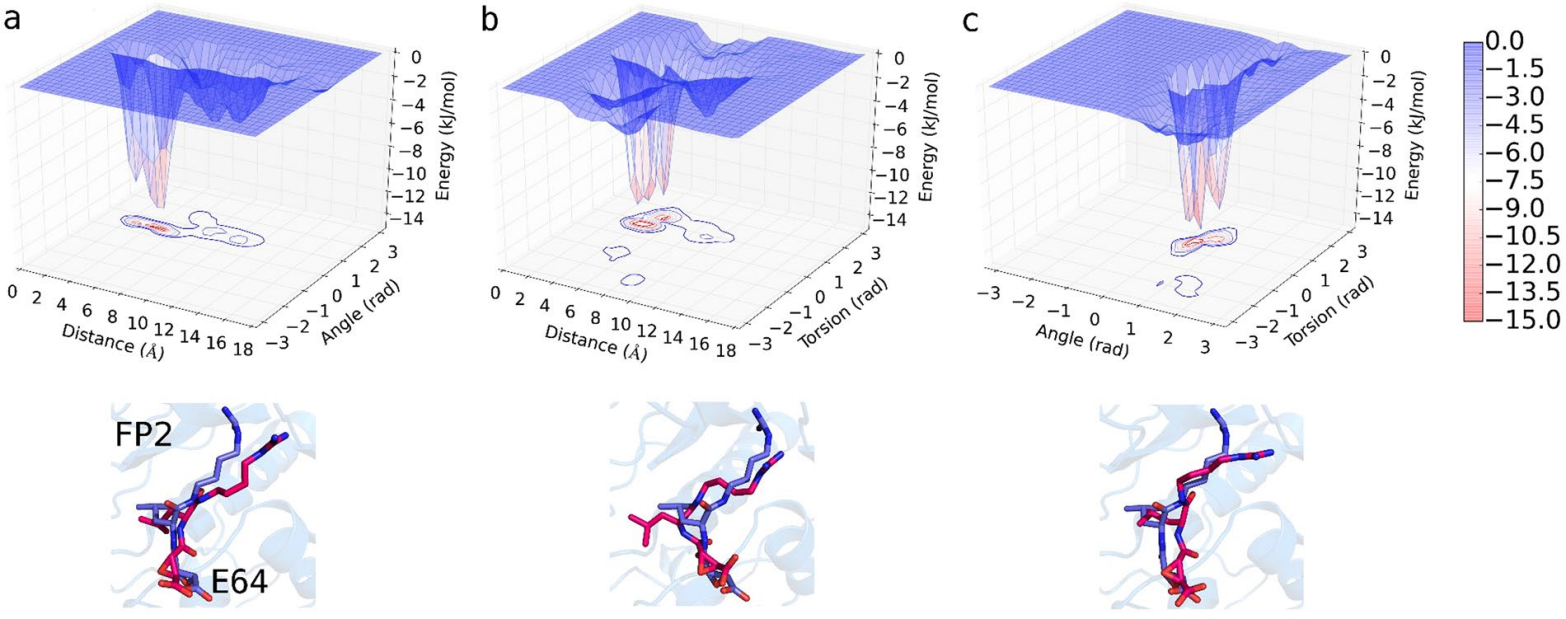
Free energy change accompanying the binding of E64 to FP2. Free energy landscapes (ΔG_binding_ = −12.2 ± 1.1 kJ/mol) obtained from ABMD (**a**) using collective variable 1 *(CV1*) and CV2, (**b**) CV1 and CV3, and (**c**) CV2 and CV3 are shown. *CV1 is the distance between centres of mass (COM) of “the first 5 (E6*4*_F5) and the last 5 (E64_L5) heavy atoms of E64” and “the heavy atoms of G83 and N173 of FP*2*”*. *CV2 is the angle between the COMs of E*6*4_F5*, *E64_L5*, *and “the heavy atoms of G83”*. *CV3 is the torsion angle between COMs of E64_F5*, *E64_L5*, *“the heavy atoms of G83”*, *and “the heavy atoms of N1*73*”*. Sample positions and conformations of E64 corresponding to the energy minima obtained from each of the three systems are shown in red (using sticks representation) below each energy landscape. The conformation of E64 in the X-ray structure is depicted in blue (using sticks representation). The positions and conformations of the E64 at the energy minima in the systems shown in (a), (b), and (c) have RMSD of 3.19 Å, 3.08 Å, and 2.90 Å from the E64 derived from the X-ray respectively.

From our adaptively biased molecular dynamics (ABMD)^27^ simulations’ results, the Gibbs free energy change accompanying the binding of E64 to FP2 is approximately −12.2 ± 1.1 kJ/mol based on the three sets of reaction coordinates/collective variables (CVs, Fig. 3a–c) used. Indeed, the ΔG_binding_ = −12.2 ± 1.1 kJ/mol reflects a strong binding and is comparable to the typical ΔG_binding_ that accompanies strong protein-ligand binding^28–31^. Please, refer to the methods section and to the caption of Fig. 3 (as well as)^32^ for more details on how the CVs were defined. The ABMD simulations reveal the regions of FP2 where E64 most favourably binds and interacts. Notable of such FP2’s regions are when CV1 = 3.8–5.9 Å & CV2 = 0.1–0.8 rad (Fig. 3a), when CV1 = 3.5–4.8 Å & CV3 = 0.7–1.0 rad (Fig. 3b), and when CV2 = 0.4–0.8 rad & CV3 = 0.3–0.7 rad (Fig. 3c). At these regions of FP2, E64 was found to interact favourably with N173, D170, H174, S149, S205, L172, N38, N81, and N86 of FP2. Furthermore, the positions and conformations of E64 at the energy minima obtained are comparable to those from the X-ray structure (with RMSD of 3.19 Å, 3.08 Å, and 2.90 Å for systems described in Fig. 3a–c respectively). It may not be possible to obtain a smaller deviation (RMSD) from the E64 in the X-ray structure as the E64 in the X-ray structure is covalently bonded to S atom of C42 of FP2, while the E64 molecule used in the ABMD simulations is not covalently bonded to FP2.

**Figure 4:**
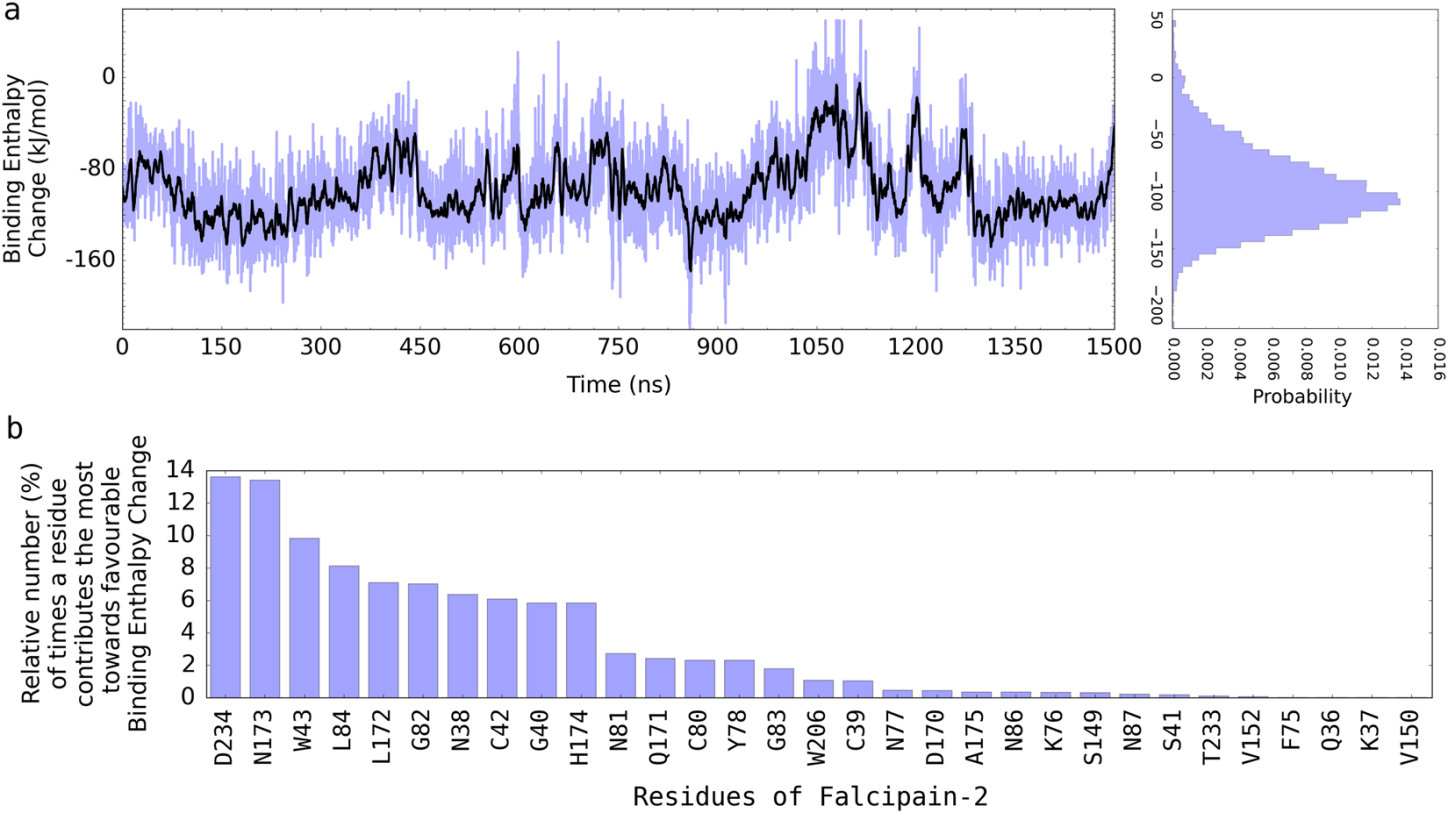
FP2-E64 binding enthalpy change obtained from MM-PBSA. (**a**) The binding enthalpy change computed from the 1500 ns trajectory is shown in blue. Its moving average (with a sliding window of 4.0 ns) is shown in black. The MM-PBSA method is used so as to decompose the contributions of each of the residues to the overall E64-FP2 binding enthalpy. Since the entropic (TΔS = −21.42 ± 1.62) component of the ΔG from MM-PBSA could not be decomposed into residue-level (i.e. residue by residue) contributions, we show only the enthalpic (ΔH) component of the ΔG in this figure which is used for comparing the relative contributions of the residues to the ΔH. The higher magnitude of the binding enthalpy or binding energy (if the effect of entropy, TΔS, is included) obtained from the MM-PBSA (compared to the results obtained from the ABMD calculations) is a known issue^32,35,36^ but does not affect the assessment of the relative contributions of each of the residues which is the sole purpose for which this method is used in this study. (**b**) A list of the residues contributing the most towards the favourable binding of E64 to FP2 are shown in (**b**).

In addition to using ABMD simulations approach for calculating the Gibbs free energy change associated with the binding of E64 to FP2, we further used Molecular Mechanics/Poisson-Boltzmann Surface Area (MM-PBSA)^33,34^ approach to assess the independent contributions of each of the residues of FP2 to the favourable binding of E64 to FP2 (Fig. 4a). (The 1500 ns trajectory obtained from the atomistic unbiased MD simulations was used for the MM-PBSA binding free energy change calculations). The MM-PBSA method, although less rigorous than the ABMD simulations approach that is used for calculating ΔG in this study, has the advantage of allowing one to be able to isolate/identify the contributions of each residue to the overall binding enthalpy change. This is the only reason for the additional use the MM-PBSA method in this study. Since the entropic (TΔS = −21.42 ± 1.62) component of the ΔG from MM-PBSA could not be decomposed into residue-level (i.e. residue by residue) contributions, we show only the enthalpic (ΔH) component of the ΔG in Fig. 4 which is used only for the purpose of comparing the relative contributions of the residues to the ΔH. Some residues stood out by contributing more than the other residues towards the binding enthalpy change. The most important contributors to the favourable binding of E64 to FP2 were found to be D234, L172, N173, W43, N38, N173, H174, L84, D170A etc. A more detailed account of these important contributors is shown in Fig. 4b and Table 2. The relatively higher magnitude of the binding energy (ΔG = ΔH − TΔS) obtained from the MM-PBSA (compared to the results obtained from the ABMD simulations approach) is a known issue^32,35,36^, but it generally does not affect the assessment of the relative contributions of each of the residues which is the sole purpose for which this method is used in this study.

**Table 2:** Effects of *in silico* mutations of selected important residues of FP2 on the E64-FP2 binding Gibbs free energy change.

	Binding ΔG* (kJ/mol)	Mutation Binding ΔΔG (kJ/mol)	A: Rank/Order Based on ΔΔG	B: Rank/Order Based on Fig. 4b	C: Combined Rank (C = A + B)
Wild Type	−12.2 ± 1.1	—	—	—	—
D234A	−0.4 ± 0.5	11.8 ± 0.9	2	1	3
*N173L*	*−0*.*7* ± *1*.*0*	*11*.*5* ± *1*.*0*	*4*	*2*	*6*
*W43F*	*−1*.*3* ± *0*.*2*	10.*9* ± *0*.*2*	*7*	3	*10*
*H174F*	*−0*.*5* ± *0*.*7*	*11*.*7* ± *0*.*7*	*3*	10	13
N38A	−0.8 ± 1.1	11.4 ± 1.1	6	7	13
L172A	−1.4 ± 0.8	10.8 ± 1.0	8	5	13
W43A	−2.5 ± 1.2	9.7 ± 1.1	10	3	13
*D234L*	*−3*.*3* ± *0*.*7*	*8*.*9* ± *0*.*7*	12	1	13
*N38L*	*−3*.*1* ± *0*.*8*	*9*.*1* ± *0*.*8*	11	7	18
N173A	−6.1 ± 0.6	6.1 ± 0.9	19	2	21
G40A	−3.3 ± 0.9	8.9 ± 1.0	13	9	22
*D170L*	*−0*.*7* ± *1*.*0*	*11*.5 ± *1*.*0*	*5*	19	24
H174A	−3.9 ± 1.2	8.3 ± 1.1	14	10	24
S41A	−0.2 ± 0.3	12.0 ± 0.8	1	25	26
G82A	−6.2 ± 1.6	6.0 ± 1.4	21	6	27
L84A	−8.1 ± 0.7	4.1 ± 0.9	23	4	27
D170A	−2.0 ± 0.9	10.2 ± 1.0	9	19	28
W206A	−3.9 ± 1.2	8.3 ± 1.1	15	16	31
N81A	−6.2 ± 1.5	6.0 ± 1.3	20	11	31
N77A	−4.3 ± 1.1	7.9 ± 1.1	17	18	35
*N81L*	*−8*.*6* ± *2*.*1*	*3*.*7* ± *2*.*1*	24	11	35
G83A	−7.4 ± 2.6	4.8 ± 2.0	22	15	37
S149A	−4.0 ± 0.5	8.2 ± 0.8	16	23	39
N86A	−4.4 ± 1.1	7.8 ± 1.1	18	21	39

^*^Each of the binding ΔG was obtained from ABMD as described in the methods section. *In silico* mutations to non-Alanine residues (e.g. *N173L* and *D234L*) are show in italics.

Furthermore, we observed that the strategic location of residues N81, L84, D170, Q171, N173 and H174, and the polarity of the side chains of residues Q171, N173, S149, S41, N77, N38, N81, N86 etc. as well as the charged nature of the side chains of H174, D170, and D234 make them very important in enhancing the interactions between E64 and FP2. These results suggest that asparagine, aspartic acid, and serine are very important in the binding of E64 to FP2 and their roles must be carefully considered when developing E64-based/E64-like antimalarial drugs to block FP2.

#### Dynamic Interactions between E64 and FP2

**Figure 5:**
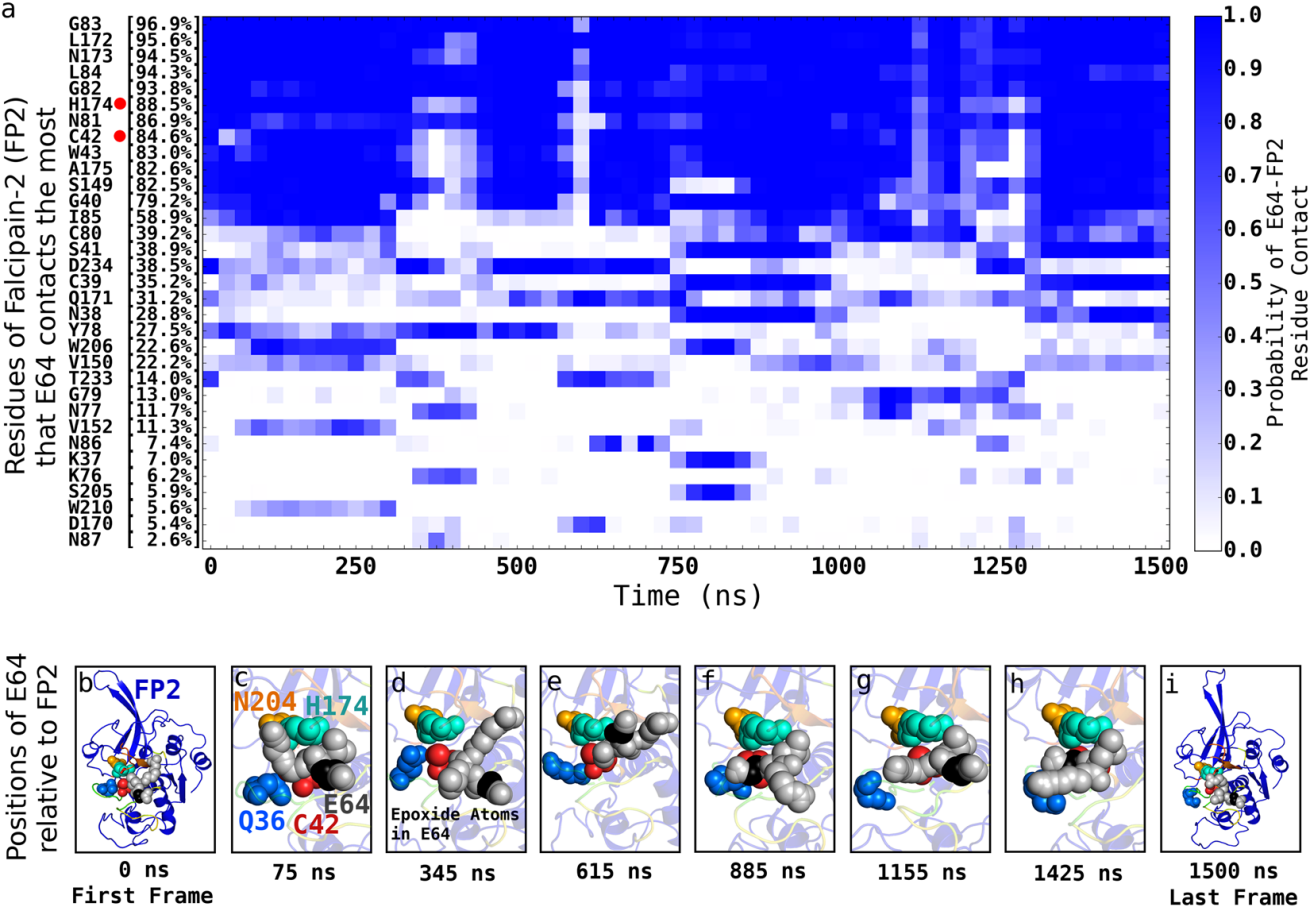
Dynamic interactions between E64 and FP2 prior to E64-FP2 covalent bond formation. The various residues of FP2 that E64 interacts with/contacts the most, and the respective residue-ligand contact profile over the 1500 ns trajectory are shown in (**a**). Each block is a bin derived from 25.0 ns segment (i.e. 1250 frames) of the trajectory. This allows the contact probability to be calculated for each block. For example, the first block for H174 shows that between 0 ns and 25.0 ns in the trajectory (made up by 1250 frames), E64 contacts H174 about 1100 times (out of the 1250 possible times) giving rise to a probability of ~0.9 which is depicted by the dark blue colour. For easy identification, red dots are placed next to the catalytic residues present in panel (a). Examples of poses of E64 relative to FP2 showing E64 persistently blocking the catalytic residues of FP2 (H174, C42, N204, and Q36, which are labelled in panel c) even prior to the formation of a covalent bond between E64 and C42 of FP2 are shown in (**b**–**i**). Please, see Supplementary Video S1 for more details.

**Figure 6:**
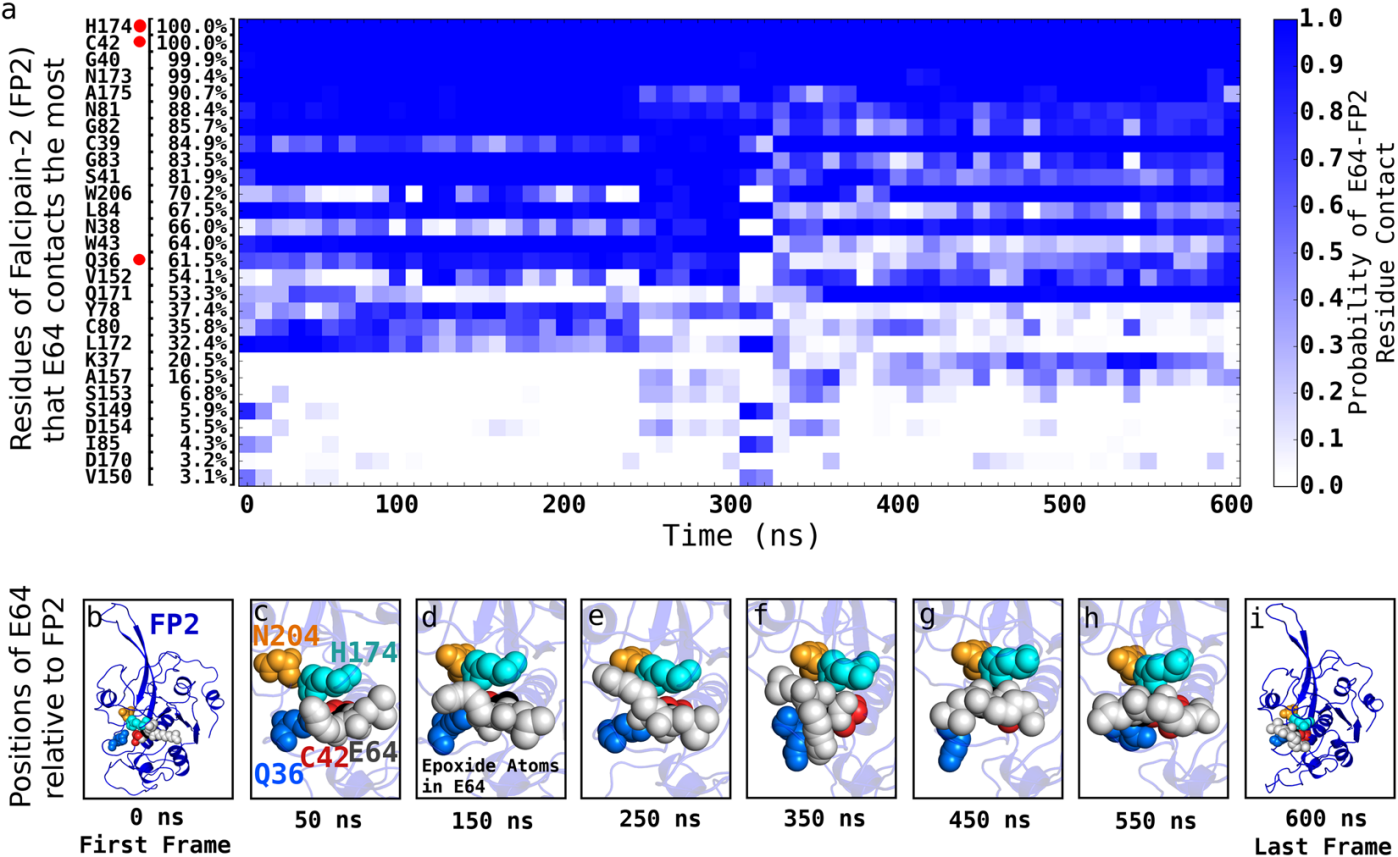
Dynamic interactions between E64 and FP2 after E64-FP2 covalent bond formation. The various residues of FP2 that E64 interacts with/contacts the most, and the respective residue-ligand contact profile over the 600 ns trajectory of a molecular system wherein E64 is covalently bonded to FP2 at the sulphur atom of CYS42 are shown in (**a**). Each of the blocks for the different residues is defined in the same way the blocks are defined in Fig. 5a. For easy identification, red dots are placed next to the catalytic residues present in panel (a). Examples of poses of E64 relative to FP2 showing E64 persistently blocking the catalytic residues of FP2 (H174, C42, N204, and Q36, which are labelled in panel c) after the formation of a covalent bond between E64 and C42 of FP2 are shown in (**b**–**i**). Please, see Supplementary Video S2 for more details.

Our 1500 ns atomistic unbiased MD simulations of the interactions between E64 and FP2 when E64 has attached to FP2 but has not yet formed covalent bond with the sulphur atom of amino acid C42 of FP2 (Fig. 5, and Supplementary Video S1) and our 600 ns atomistic unbiased MD simulations of the interactions between E64 and FP2 after the covalent bond has been formed (Fig. 6, and Supplementary Video S2) show that, in addition to interacting with the amino acid residues lining the established binding pocket subsites (of FP2), E64 also frequently interacts with some residues outside the established subsites of FP2’s binding pocket (Table 1, Fig. 2). At this point, we must emphasise that we also examined the topology of the surface of FP2 with Computed Atlas of Surface Topography of proteins (CASTp)^37,38^. The binding pockets of FP2 suggested by CASTp is bigger than and involves more residues than the previously established binding pocket subsites^39,40^. We found the S1, S2, S1’, and S3 definition of FP2’s binding pocket subsites^39,40^ to be more informative, and use it when referring to the binding pockets of FP2 in this work. Furthermore, we found that outside the established binding pocket’s subsites (S1, S2, S1’, and S3), E64 frequently (i.e. up to 97% of the time) interacts with FP2’s G83 (97%), N173 (95%), L84 (94%), H174 (89%), N81 (87%), C42 (85%), A175 (83%), S149 (83%), D234 (39%), etc. (Fig. 5a). The nature of the overall interactions between E64 and the residues of FP2 are summarised in Fig. 5a. The positions of some of these residues are already shown in Fig. 2a.

#### The FP2 Binding Pocket Residues and the Nearby Residues are Important to the Stability of the FP2-E64 Complex

From the 1500 ns trajectory of the interactions between E64 and FP2 (Fig. 5a, Supplementary Video S1), and the MM-PBSA results (Fig. 4a, Table 1), we found that the residues lining the binding pocket’s subsites of FP2 are involved in only a fraction of the interactions between E64 and FP2, and that a number of FP2’s residues outside (but nearby) the established binding pocket of FP2 (such as D170, A175, H174, A151, N173, G79, Q171, A44, S205, and I148) are involved in a large fraction of the interactions between E64 and FP2 both prior to (Fig. 5, Supplementary Video S1) and after (Fig. 6, Supplementary Video S2) the formation of a covalent bond between E64 and FP2. These suggest that E64’s interactions with residues outside the binding pocket also contribute considerably to the stability of the FP2-E64 complex. In fact, their *in silico* mutations resulted in considerable weakening of FP2-E64 binding (Table 2).

In addition, we found that binding free energy change contributions from only the residues lining the previously established FP2’s binding pocket subsites are able to explain only 45.3% of the stability of the FP2-E64 complex (Supplementary Table S2), while the contributions from residues nearby the binding pocket’s subsites are able to explain 59.8% of the stability of the FP2-E64 complex (Supplementary Table S3). These suggest the need to carefully consider the roles of the residues surrounding the binding pocket (and not just those lining the binding pocket) when designing drugs to block the activities of FP2, but with a caution that the developed drug should still bind strongly to FP2 at its active site or (at least) near enough to FP2’s active site to block the catalytic residues of FP2. Furthermore, these results suggest that the initial characterization of the binding pocket of FP2^39,40^ may need to be reviewed and revised.

Our further investigations revealed that hydrogen bonding, electrostatic interactions, and van der Waals interactions between E64 and FP2’s (binding and non-binding pockets’) residues are able to explain the binding Gibbs free energy change of FP2-E64 complex. We provide a summary of how hydrogen bonds, and electrostatic interactions, as well as van der Waals interactions between E64 and specific residues of FP2 contribute towards the binding of E64 to FP2 in Table 1. Furthermore, we show (in Supplementary Table S4) that apart from the direct hydrogen bonding between E64 and the residues of FP2, water molecules play additional vital roles in the binding between E64 and FP2 by serving as “bridges” for a lot of E64-FP2 hydrogen bonding.

### E64 Persistently Blocks the Catalytic Residues of FP2

With a binding Gibbs free energy change of −12.2 ± 1.1 kJ/mol (Fig. 3a–c), the E64’s persistent binding to FP2 is energetically favourable and stable. We show in Fig. 5c,d (as well as in Supplementary Video S1) typical situations where three catalytic residues of FP2 (C42, H174, and N204) are blocked altogether by E64 at a time in a system where E64 has not yet formed a covalent bond with FP2. Similar typical situations where all the catalytic residues of FP2 are blocked all together at a time in the system in which E64 has already formed a covalent bond with FP2 are shown in Fig. 6e–h (as well as in Supplementary Video S2).

The strategic (place of) binding of E64 to FP2 (Fig. 5b–i) whereby E64 persistently blocks one or more catalytic residues of FP2 (Fig. 5b–i) in addition to the stability of the binding of E64 to FP2 (Fig. 1f, Fig. 2a) allows E64 to be able to inhibit cysteine protease activities of FP2. For example, we found that, even before forming a covalent bond with FP2, E64 blocks FP2’s catalytic residue H174 88.5% of the time (Fig. 5a), and blocks the catalytic residue C42 84.6% of the time (Fig. 5a), etc. in a manner that all the essential catalytic residues of FP2, together as a functional unit, are never free from E64’s blockade at any point in time. In addition, the 3D structure/conformation of FP2 necessitate that N204 and/or Q36 are not (at all or not easily) accessible whenever either of or both of H174 and C42 are blocked by E64. We believe that, since the catalytic residues of FP2 must work together to achieve the catalysis, the persistent blocking of H174 and C42 by E64 (and the occlusion of the paths to the relatively buried Q36 and N204) may adequately indicate that the inhibition of FP2 by E64 is persistent. Representative structures and the positions of E64 relative to FP2’s active sites over the 1500 ns trajectory are shown in Fig. 5b–i as well as in Supplementary Video S1.

### Implications for Antimalarial Drug Design

We have extensively studied the mechanisms by which E64 approaches FP2, tightly binds to FP2, and inhibits FP2. Our findings have shown that it is not only the residues lining the four subsites (S1, S2, S1’, and S3) of the binding pocket of FP2 that are important for E64’s binding to FP2, and that the residues that are not within (but nearby) the binding pocket are also very important. These suggest that efforts to design antimalarial drugs that block cysteine protease activities of FP2 should not focus only on finding drug candidates that will bind tightly to the residues of the established binding pocket but also consider the need for such drug candidate to be able to bind to the residues immediately surrounding and/or nearby the established binding pocket subsites. More specifically, in the current study, we observed that N38, C42, N81, Q171, C39, H174, S41, W43, D170, A175, and N173, which are outside the four subsites of FP2’s binding pocket, are important for the facilitation of E64’s tight binding to FP2 and its blockage of FP2’s active site residues even prior to the formation of a covalent bond between E64 and FP2. For example, N81A, H174A, S41A, W43A, D170A, N173A, etc. (which are some of the FP2 mutations studied *in silico*) show that the *in silico* mutations of these important residues adversely affect E64-FP2 binding (Table 2).

**Figure 7:**
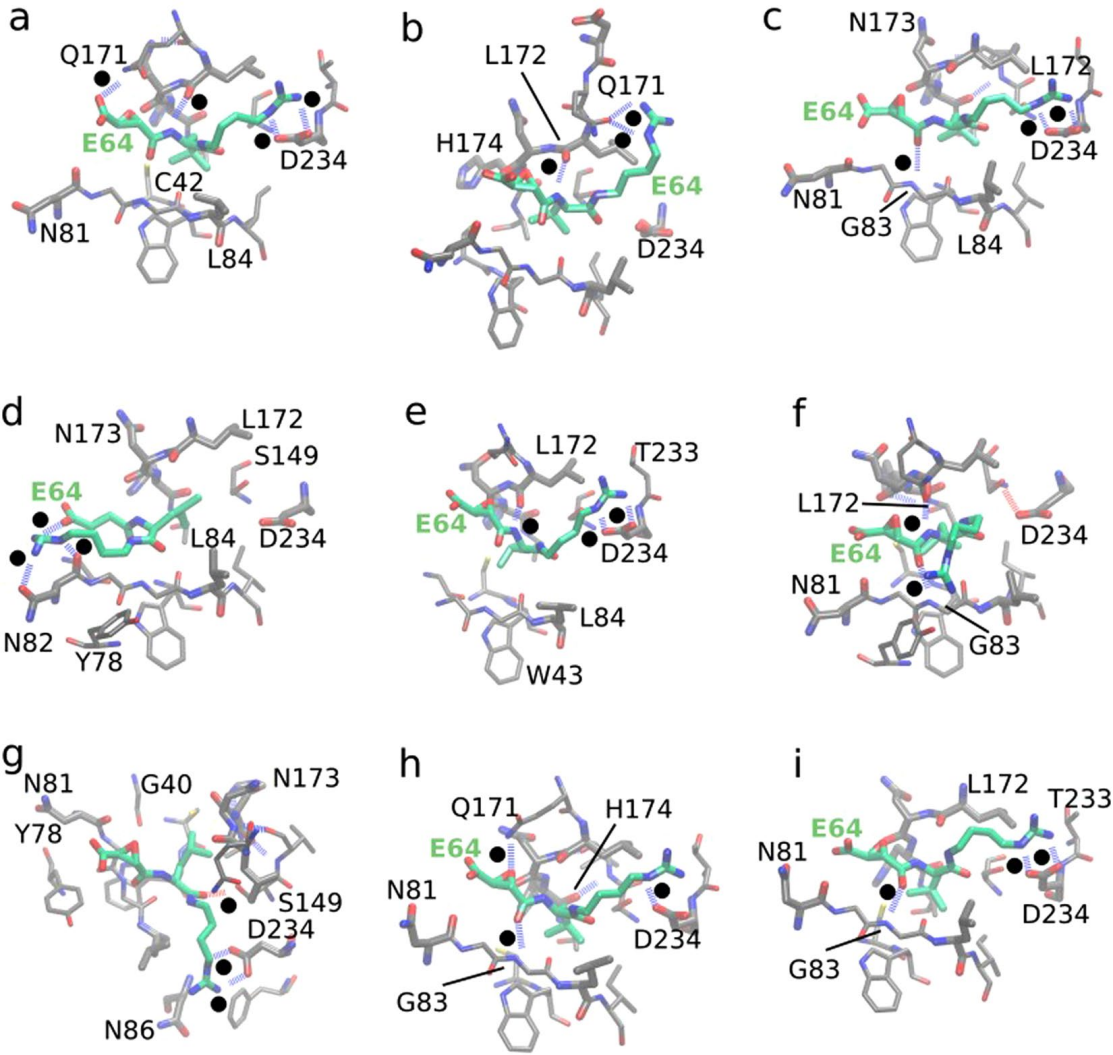
Non-bonded interactions between E64 and FP2. Examples of non-bonded interactions, most especially hydrogen bonding between E64 and FP2 are shown here: panels (a) to (i). Hydrogen bonds are represented by the broken lines. To avoid congestion, hydrogen atoms are not explicitly shown. To guide the readers’ eyes, black dots are placed next to each of the broken lines that represent the hydrogen bonds. Some of the vital roles played by water molecules, wherein water molecules form hydrogen bonds with the residues of FP2 and with some atoms of E64 thereby enhancing the trapping of E64 within the binding cavities of FP2 are shown in Supplementary Fig. 1. In each of the cases, we observed favourable van der Waals interactions between E64 and FP2 and noticed that such favourable van der Waals interactions are made possible and enhanced by the flexibility of the backbone of E64 allowing it to flexibly fit into FP2’s binding cavity with good surface complementarity as shown in Supplementary Fig. S1.

Furthermore, our findings established that hydrogen bonding, electrostatic interactions, and van der Waals interactions are the very important nonbonded interactions between E64 and FP2 facilitating the stability of the complex. We show examples of such interactions (most especially, hydrogen bonding and van der Waals interactions) in Fig. 7 and Supplementary Fig. S1. Favourable hydrogen bonding between networks of water molecules, atoms of E64, and residues of FP (such as N173, N81, L172, D234, S149, etc.) form an envelope that appears to trap E64 in the FP2’s cavity (such as shown in Supplementary Fig. S1a–d), which favours E64-FP2 binding. This is further enhanced by the hydrophobic interactions between FP2 and the non-polar part of the E64 (Fig. 7, Supplementary Fig. S1). The non-polar part of E64 (such as its carbon-cluster branch, Fig. 1e) is persistently buried deep in the FP2’s catalytic cavity nearby FP2’s active site residues (Fig. 7, Supplementary Fig. S1). In addition, E64 is observed forming several hydrogen bonds with FP2 (with and without the help of water molecules which form bridges between the ligand and the protein) as shown in Fig. 7, Supplementary Fig. S1, and Supplementary Table S4. Therefore, we believe that a potential antimalarial drug candidate targeted at blocking the activities of FP2 would benefit from simultaneously having (1) a hydrophobic part *(such as the “carbon-cluster branch” of E64*, Figs 1e and 7) which can be easily buried in the catalytic cavity of FP2 near the catalytic residues and (2) polar parts *(such as the “nitrogen-rich” and the “oxygen-rich” termini of E64*, Figs 1e and 7) that can participate in vital hydrogen bonding and electrostatic interactions.

Furthermore, the highly favourable van der Waals interactions between E64 and FP2 that is observed (Table 1) and traced back to the flexibility of E64’s backbone which allows E64 to flexibly adjust its shape to achieve favourable surface complementarity (and thus favourable van der Waals interactions) with FP2’s binding pockets (Supplementary Fig. 1) suggests that the potential E64-based/E64-like antimalarial drug targeted at blocking the activities of FP2 would (in addition to all the features already identified above) benefit from having a flexible backbone. Nonetheless, we believe that the electrostatic interactions between such E64-based/E64-like drug and FP2 would be more important (Table 1).

These suggest that potential drug candidates for blocking the activities of FP2 should have good hydrogen-bonding capability, and very importantly, good tendencies for electrostatic interactions with FP2 residues at and nearby the catalytic residues. More specifically, such drug candidates should be (1) capable of being both hydrogen bonds donor and hydrogen bonds acceptor, (2) capable of forming favourable/attractive van der Waals and electrostatic interactions with Q171, N173, S149, S41, N77, N38, N81, and N86 (which have polar of the sides chains), as well as with H174, D170, and D234 (which have charged side chains). More importantly, it remains of highest importance that the potential drug binds FP2 strongly at its active site or (at least) near enough to the active site of FP2 to block the catalytic residues of FP2.

Indeed, it is worth mentioning that our results suggest that the ability to favourably interact with asparagine (specifically, N173, N77, N38, N81, N86, and N204) and aspartic acid (specifically, D170, and D234), which are examples of amino acids with polar side chains and amino acids with negatively charged side chains respectively, might be extremely important for any potential E64-based antimalarial drug targeted at blocking FP2’s activities.

## Conclusions

*Plasmodium falciparum* malaria is a serious health problem that is still endemic in many Tropical (especially Tropical African) countries. *P*. *falciparum* uses its falcipain-2 (FP2), a cysteine protease, to degrade the host’s haemoglobin. We have shown the time-resolved mechanistic details of how E64 approaches and binds to FP2, the dynamic interactions between E64 and FP2, and the factors that enhance the favourable and stable binding of E64 to FP2 both prior to and after E64-FP2 covalent bond formation. Our results confirm that E64 is able to persistently inhibit FP2, and explain in details the physicochemical factors that make E64’s interaction with FP2 favourable. Furthermore, it is established that the residues lining the known binding pocket of FP2 (namely D234, G82, V152, K76, N77, L172, Y78, and W206) as well as some residues nearby FP2’s know binding pocket (namely N38, C42, N81, Q171, C39, H174, S41, S149, W43, D170, A175, and N173) are very important in E64’s strong binding to FP2 and E64’s persistent blocking of the catalytic residues of FP2. A molecule/potential drug with similar ability to strongly bind FP2 (even without necessarily forming a covalent bond with FP2) and persistently block the catalytic residues of FP2 could have potential antimalarial effects. Furthermore, the results of this study suggest that the ability to favourably interact with asparagine, aspartic acid, and serine might be important characteristics that a potential E64-like antimalarial drug targeted at blocking FP2’s activities should have. The results of this study have the potentials of guiding the development of antimalarial drugs that block the activities of FP2.

## Methods

### Structure of FP2 and E64

We obtained X-ray crystallographic structure of FP2-E64 complex (PDB ID: 3BPF)^25^ from the Protein Data Bank (PDB)^41^, and obtained the all-atom structure of E64 from ZINC database^42^ (ZINC ID: ZINC13493525) because the structure of E64 in the PDB (3BPF) lack hydrogen atoms.

### Force Field Parameters for E64

We used Antechamber from AmberTools16^43,44^ to create the needed E64’s force-field parameters. Density and heat of vaporization were assessed for the force field validations. We provide access to copies of the created force-field parameters and their usage online at http://bioinformatics.center/research/malaria/e64.

### Molecular Dynamics (MD) Simulations

Two explicitly solvated initial molecular systems (with TIP3P water^45^ molecules) for atomistic unbiased molecular dynamics (MD) simulations were created using tLeap from AmberTools16 and AMBER ff14SB force-fields^46^. One of the systems includes E64 attached to FP2 but not covalently bonded to FP2. This design made possible the studying of how a potential *(and*, *perhaps*, *a reversible)* antimalarial drug candidate would interact with FP2 without necessarily forming a covalent bond *(or*, *for irreversible inhibition*, *before forming a covalent bond)* with the target. In other words, the studying of the non-covalently bonded E64-FP2 system allowed us to investigate (1) how E64 approaches FP2, (2) how E64 interacts with FP2 prior to forming covalent bond with it, and (3) the binding energy involved in the E64-FP2 complex even prior to or without the formation of a covalent bond between E64 and FP2. The second system is also made up by E64 and FP2 but, therein, the E64 is covalently bonded to FP2. In other words, the entire study was essentially modelled as a two-stage process (namely, before and after the formation of a covalent bond between E64 and FP2) and the forcefields for each of the two stages were parameterised as discussed in the previous subsection.

The systems were energy minimised using AMBER16^43,44,47^. The energy minimizations were done in three stages – weakly (0.5 kcal/mol/Å^2^) restraining all non-water atoms in the first stage, all alpha carbon (CA) atoms and all heavy atoms of E64 in the second stage, and without any restraints in the third stage. While putting weak restraints (0.5 kcal/mol/Å^2^) on CA atoms and the heavy atoms of E64, each of the systems was gradually heated to 310 K in canonical ensemble, and the system’s temperature was maintained at 310 K during the equilibration and production runs. Following equilibration, we carried out 1500 ns of unrestrained and unbiased MD simulations in isothermal-isobaric (NPT) ensemble for the system without a covalent bond between E64 and FP2, and 600 ns for the system where E64 is covalently bonded to FP2. Langevin thermostat with a collision frequency of 2 ps^−1^ was used for temperature control. Berendsen barostat^48^ was used for pressure control. We calculated full electrostatic interactions energies by Particle Mesh Ewald method^49^, and used a cut-off distance of 10 Å for non-bonded interactions.

### MD Simulations Study of How E64 Approaches and Binds to FP2

To study how E64 approaches and binds to FP2, we created 50 explicitly solvated systems (with TIP3P water^45^ molecules) wherein E64 is placed 15 Å from FP2 (from the side of FP2 where E64 is expected to bind) but with very slight differences in the orientations of the E64 across the 50 systems. We energy minimised the system (first, with weak restraints, 0.5 kcal/mol/Å^2^, on all non-water atoms; second, on all CA atoms and all the heavy atoms of E64; and finally without any restraints). The systems were then gradually heated to 310 K with weak restraints, 0.5 kcal/mol/Å^2^, all CA atoms and all the heavy atoms of E64 followed by the production atomistic unbiased MD simulations at 310 K without any restraints. For each of the 50 replicates, up to 75 ns trajectory was generated for the production unrestrained unbiased MD simulations run giving rise to (75 ns * 50 = 3,750 ns) 3.75 microseconds MD simulations trajectory on how E64 approaches FP2. The positions visited by E64 relative to FP2 were tracked in each of the trajectories. The results (presented in Fig. 2) show how E64 approaches and binds to FP2.

### Binding Free Energy Change Calculations by ABMD

**Figure 8:**
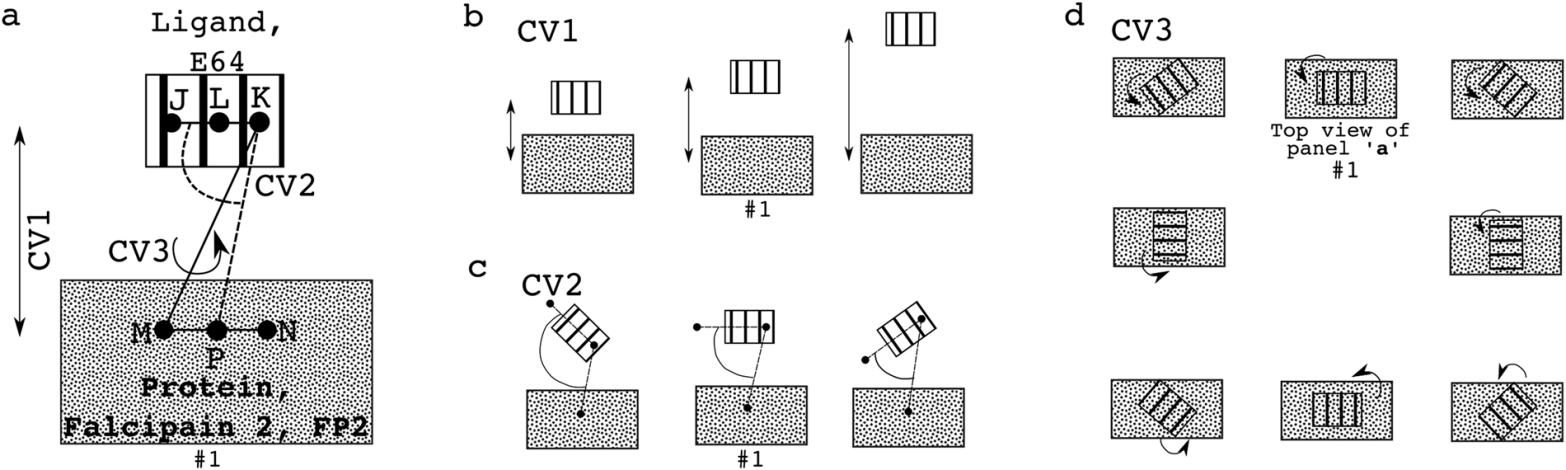
Collective variables (CVs) for calculating the binding Gibbs free energy change through ABMD. In each of the sub-figures, the smaller structure (with stripes) represents E64 (the ligand), while the bigger structure (with dotted grey shades) represents the FP2 (the protein). (**a**) The three CVs/reaction coordinates used for defining the three pairs of CVs are shown as CV1, CV2, and CV3. CV1 is the distance between L and P. L is the centre of mass of J and K, while and P is the centre of mass of M and N. CV2 is the angle formed by J, K, and P. CV3 is the torsion angle formed by J, K, M, and N. Examples of changes in the position of the E64 relative to FP2 when CV1 is sampled/explored is shown in (**b**); when CV2 is sampled is shown in (**c**); when CV3 is sampled is shown in (**d**). Panel (d) is made up by views from the top so as to easily demonstrate the effects of changes in the torsional angle (CV3) on the relative orientations of E64 to FP2. In each of the panels (a to d), the setup numbered “#1” depicts the initial E64-FP2 relative orientation before CV1, CV2, and/or CV3 is(are) explored^32^.

Three sets of collective variables (CVs, Fig. 8a) were used^32^ for the adaptively biased molecular dynamics (ABMD) simulations^27,50–52^: (1) “CV1 and CV2”, (2) “CV1 and CV3”, and (3) “CV2 and CV3”. CV1 is a distance-based CV (Fig. 8a,b), CV2 is an angle-based CV (Fig. 8a,c), and CV3 is a torsion-angle-based CV (Fig. 8a,d). Each of the ABMD simulations was run until the ΔG converged.

### Binding Free Energy Change Calculations by MM-PBSA

In addition to the ABMD simulations approach, the binding free energy change was calculated for each frame from the 1500 ns production MD simulations trajectory using Molecular Mechanics Poisson-Boltzmann Surface Area (MM-PBSA)^33,34,53^ implemented in AmberTools16. The binding free energy (Δ*G*_*bind*_) accompanying the complexation of a receptor and a ligand to form a complex can be estimated from MM-PBSA as

1 $${{\rm{\Delta }}{\rm{G}}}_{{\rm{binding}}}={\rm{\Delta }}{\rm{H}}-{\rm{T}}{\rm{\Delta }}{\rm{S}}\approx {{\rm{\Delta }}{\rm{E}}}_{{\rm{MM}}}+{{\rm{\Delta }}{\rm{G}}}_{{\rm{sol}}}-{\rm{T}}{\rm{\Delta }}{\rm{S}}$$

where Δ*E*_*MM*_, Δ*G*_*sol*_ and −*TΔS* are respectively gas-phase Molecular Mechanics (MM) energy change, solvation free energy change, and conformational entropy change upon binding. While −*T*Δ*S* can be computed by normal-mode analysis, ΔE_MM_ and ΔG_sol_ can be expressed as follows.

2 $${{\rm{\Delta }}{\rm{E}}}_{{\rm{MM}}}={{\rm{\Delta }}{\rm{E}}}_{{\rm{internal}}}+{{\rm{\Delta }}{\rm{E}}}_{{\rm{electrostatics}}}+{{\rm{\Delta }}{\rm{E}}}_{{\rm{vdw}}}$$

3 $${{\rm{\Delta }}{\rm{G}}}_{{\rm{sol}}}={{\rm{\Delta }}{\rm{G}}}_{{\rm{PB}}}+{{\rm{\Delta }}{\rm{G}}}_{{\rm{SA}}}$$

where *ΔG*_*PB*_ is (the polar contribution to) electrostatic solvation energy calculated using Poisson Boltzmann (PB) model. *ΔG*_*SA*_ is (the non-electrostatic contributions to) the solvation component estimated by solvent accessible surface area.

The MM-PBSA method, although less rigorous than the ABMD simulations approach that is also used for calculating ΔG in this study, has the advantage of allowing one to be able to isolate/identify the contributions of each residue to the overall binding energy/enthalpy change. This is the sole reason for using the MM-PBSA method in this study.

### Ligand-Residue Contact

We recorded a contact between E64 and a given residue of FP2 whenever any heavy atom of E64 is within 5.0 Å of any heavy atom in a given residue/amino acid of FP2.

### Hydrogen Bonds

A hydrogen bond is recorded between two units whenever a hydrogen-bond acceptor (HBA) of one of the units is within 3.5 Å of a hydrogen-bond donor (HBD) of the other unit and the angle formed by HBA-Hydrogen-HBD is greater than or equal to 120°.

## Electronic supplementary material

Supplementary information

E64 binds to Falcipain-2, and inhibits its catalytic residues

E64 binds to Falcipain-2 and irreversibly blocks its catalytic residues

## Data Availability

The initial structure of falcipain-2-E64 complex is available in the Protein Data Bank (PDB ID: 3BPF). Other dataset (such as Trajectories from Molecular Dynamics Simulations, etc.) upon which the results presented in the paper are based can be requested from the corresponding author or from Bioinformatics Center (tools@Bioinformatics.Center).
