# Supplementary material for: In Silico Study Reveals How E64 Approaches, Binds to, and Inhibits Falcipain-2 of Plasmodium falciparum that Causes Malaria in Humans
Source: Sci Rep. 2018 Nov 6;8:16380. doi: 10.1038/s41598-018-34622-1 (PMC6219542; doi:10.1038/s41598-018-34622-1)
Supplement: Supplementary file 1 — Supplementary information [file 41598_2018_34622_MOESM1_ESM.doc]

**Title: *In Silico* Study Reveals How E64 Approaches, Binds to, and Inhibits Falcipain-2 of *Plasmodium falciparum* that Causes Malaria in Humans**

**Running Title: E64 Blocks the Active Site of Falcipain-2 of Malaria Protozoan Parasites**

Emmanuel Oluwatobi Salawu ^1,2,3,4,5^

ORCID: 0000-0002-4977-0917

1 Insilico Taiwan, Taipei City, Taiwan

2 TIGP Bioinformatics Program, Academia Sinica, Taipei City, Taiwan

3 Institute of Bioinformatics and Structural Biology, National Tsing Hua University, Hsinchu City, Taiwan

4 School of Computer Science, University of Hertfordshire, Hatfield, United Kingdom

5 Bioinformatics Center, Sheridan, WY, USA

**ADDRESS FOR CORRESPONDENCE**

Emmanuel Oluwatobi Salawu, Institute of Bioinformatics and Structural Biology, National Tsing Hua University, Hsinchu, Taiwan; emmanuel@gapp.nthu.edu.tw; +886 905290930

**CONFLICT OF INTERESTS/COMPETING INTERESTS**

There are no financial and/or non-financial competing interests whatsoever.

**SUPPLEMENTARY INFORMATION**

**Supplementary Video S1:** Dynamic interactions between E64 and FP2 shows how E64 *persistently blocks* the catalytic residues of FP2 even before E64-FP2 covalent bond formation: <https://goo.gl/K1Txks>

**Supplementary Video S2:** Dynamic interactions between E64 and FP2 shows how E64 *irreversibly blocks* the catalytic residues of FP2 after E64-FP2 covalent bond formation: <https://goo.gl/udBXJg>

**Supplementary Table S1:** Regression model showing the contributions of hydrogen bonds, electrostatic, and van der Waals interactions towards the favourable binding of E64 to FP2

**Supplementary Table S2:** Contributions of the residues lining the established FP2's binding pocket subsites towards the favourable binding of E64 to FP2

**Supplementary Table S3:** Contributions of the residues outside the established FP2's binding pocket subsites towards the favourable binding of E64 to FP2

**Supplementary Fig. S1:** Hydrogen bonding and other non-bonded interactions between E64 and FP2

**Supplementary Table S4:** List of residues of FP2 forming hydrogen bonds with E64 through hydrogen bridges created by water molecules

**Supplementary Table S1:** Regression model showing the contributions of hydrogen bonds, electrostatic, and van der Waals interactions towards the favourable binding of E64 to FP2

OLS Regression Results

==============================================================================

Dep. Variable: energy R-squared: 0.679

Model: OLS Adj. R-squared: 0.673

Method: Least Squares F-statistic: 110.9

No. Observations: 4493 AIC: 3.494e+04

Df Residuals: 4408 BIC: 3.548e+04

Df Model: 84 Prob (F-statistic): 0.00

Covariance Type: nonrobust Log-Likelihood: -17383.

===============================================================================

coef std err t P>|t| [95.0% Conf. Int.]

-------------------------------------------------------------------------------

Intercept 15.6211 1.443 10.824 0.000 12.792 18.450

ASP_234 -5.5442 1.821 -3.045 0.002 -9.114 -1.974

ASN_173 0.5644 0.531 1.062 0.288 -0.477 1.606

LEU_172 -1.4229 0.831 -1.713 0.087 -3.051 0.205

ASN_38 -49.9639 12.478 -4.004 0.000 -74.427 -25.501

LEU_84 0.6873 0.342 2.011 0.044 0.017 1.357

HIS_174 -3.0636 0.566 -5.410 0.000 -4.174 -1.953

GLY_82 -3.0002 0.836 -3.590 0.000 -4.639 -1.362

TRP_43 -0.4500 0.765 -0.588 0.557 -1.950 1.050

TYR_78 2.7656 2.459 1.125 0.261 -2.056 7.587

GLN_171 -5.1885 1.838 -2.822 0.005 -8.793 -1.584

CYS_42 -11.3194 2.235 -5.064 0.000 -15.702 -6.937

GLY_40 -3.2700 1.594 -2.052 0.040 -6.395 -0.145

GLY_83 0.0970 0.978 0.099 0.921 -1.820 2.014

TRP_206 18.7347 11.822 1.585 0.113 -4.443 41.912

ASN_81 -10.3026 1.650 -6.243 0.000 -13.538 -7.067

CYS_80 -1.182e-13 1.33e-13 -0.890 0.373 -3.79e-13 1.42e-13

ALA_175 0.2124 0.895 0.237 0.812 -1.542 1.966

CYS_39 -3.6442 0.997 -3.654 0.000 -5.599 -1.689

ASN_77 -2.0103 2.754 -0.730 0.465 -7.409 3.388

SER_149 -4.24e-13 2.24e-13 -1.893 0.058 -8.63e-13 1.5e-14

ASP_170 3.909e-13 1.17e-13 3.343 0.001 1.62e-13 6.2e-13

ASN_87 2.7089 2.213 1.224 0.221 -1.630 7.048

LYS_76 -1.1825 2.320 -0.510 0.610 -5.731 3.365

ASN_86 -0.8020 0.860 -0.932 0.351 -2.489 0.885

SER_41 -3.1526 0.903 -3.492 0.000 -4.923 -1.383

THR_233 1.878e-13 1.06e-13 1.765 0.078 -2.08e-14 3.96e-13

PHE_75 -6.0640 6.929 -0.875 0.382 -19.648 7.520

VAL_152 -4.437e-15 6.59e-14 -0.067 0.946 -1.34e-13 1.25e-13

GLN_36 4.215e-14 4.33e-14 0.974 0.330 -4.27e-14 1.27e-13

LYS_37 4.9921 6.006 0.831 0.406 -6.783 16.767

ASP_234_ELE 0.0866 0.008 11.347 0.000 0.072 0.102

ASN_173_ELE 0.0399 0.023 1.754 0.079 -0.005 0.084

LEU_172_ELE 0.3788 0.033 11.603 0.000 0.315 0.443

ASN_38_ELE 0.1759 0.031 5.747 0.000 0.116 0.236

LEU_84_ELE 0.8933 0.117 7.636 0.000 0.664 1.123

HIS_174_ELE 0.7609 0.050 15.071 0.000 0.662 0.860

GLY_82_ELE 0.2203 0.049 4.482 0.000 0.124 0.317

TRP_43_ELE 0.2642 0.087 3.022 0.003 0.093 0.436

TYR_78_ELE 0.1258 0.023 5.518 0.000 0.081 0.171

GLN_171_ELE 0.1729 0.022 7.973 0.000 0.130 0.215

CYS_42_ELE 0.3398 0.053 6.364 0.000 0.235 0.444

GLY_40_ELE 0.3982 0.060 6.600 0.000 0.280 0.516

GLY_83_ELE 0.9687 0.048 20.270 0.000 0.875 1.062

TRP_206_ELE 0.4243 0.055 7.749 0.000 0.317 0.532

ASN_81_ELE 0.2296 0.022 10.312 0.000 0.186 0.273

CYS_80_ELE -0.1190 0.059 -2.023 0.043 -0.234 -0.004

ALA_175_ELE -1.4159 0.158 -8.958 0.000 -1.726 -1.106

CYS_39_ELE 0.2730 0.047 5.837 0.000 0.181 0.365

ASN_77_ELE 0.2804 0.077 3.649 0.000 0.130 0.431

SER_149_ELE 0.0362 0.044 0.826 0.409 -0.050 0.122

ASP_170_ELE 0.0263 0.016 1.651 0.099 -0.005 0.057

ASN_87_ELE -0.2583 0.074 -3.502 0.000 -0.403 -0.114

LYS_76_ELE 0.1598 0.023 6.981 0.000 0.115 0.205

ASN_86_ELE 0.0831 0.052 1.611 0.107 -0.018 0.184

SER_41_ELE 0.3054 0.080 3.823 0.000 0.149 0.462

THR_233_ELE -0.3446 0.059 -5.825 0.000 -0.461 -0.229

PHE_75_ELE 0.5858 0.100 5.834 0.000 0.389 0.783

VAL_152_ELE 0.0187 0.103 0.181 0.856 -0.183 0.221

GLN_36_ELE 0.7645 0.076 10.065 0.000 0.616 0.913

LYS_37_ELE -0.3344 0.033 -10.228 0.000 -0.399 -0.270

ASP_234_vDW -0.4957 0.085 -5.840 0.000 -0.662 -0.329

ASN_173_vDW 0.0008 0.080 0.010 0.992 -0.156 0.158

LEU_172_vDW 0.7539 0.098 7.677 0.000 0.561 0.946

ASN_38_vDW 0.5090 0.182 2.794 0.005 0.152 0.866

LEU_84_vDW 0.1325 0.107 1.235 0.217 -0.078 0.343

HIS_174_vDW 0.1177 0.122 0.966 0.334 -0.121 0.357

GLY_82_vDW 0.5037 0.149 3.380 0.001 0.212 0.796

TRP_43_vDW 0.9540 0.148 6.425 0.000 0.663 1.245

TYR_78_vDW 0.8068 0.099 8.131 0.000 0.612 1.001

GLN_171_vDW 0.9187 0.127 7.230 0.000 0.670 1.168

CYS_42_vDW 0.2549 0.154 1.658 0.097 -0.046 0.556

GLY_40_vDW 0.6423 0.144 4.475 0.000 0.361 0.924

GLY_83_vDW 0.5436 0.102 5.317 0.000 0.343 0.744

TRP_206_vDW 0.1361 0.182 0.748 0.455 -0.221 0.493

ASN_81_vDW 0.0666 0.114 0.584 0.559 -0.157 0.290

CYS_80_vDW 0.0641 0.276 0.232 0.816 -0.477 0.605

ALA_175_vDW 0.3932 0.230 1.710 0.087 -0.058 0.844

CYS_39_vDW 0.6940 0.218 3.191 0.001 0.268 1.120

ASN_77_vDW 1.7675 0.354 4.998 0.000 1.074 2.461

SER_149_vDW 0.2807 0.155 1.812 0.070 -0.023 0.584

ASP_170_vDW 0.8092 0.369 2.190 0.029 0.085 1.534

ASN_87_vDW 0.1230 0.621 0.198 0.843 -1.094 1.340

LYS_76_vDW 1.7849 0.446 4.004 0.000 0.911 2.659

ASN_86_vDW -2.8127 0.386 -7.292 0.000 -3.569 -2.056

SER_41_vDW -0.6959 0.519 -1.342 0.180 -1.713 0.321

THR_233_vDW 0.4659 0.261 1.786 0.074 -0.046 0.978

PHE_75_vDW 0.8898 0.400 2.223 0.026 0.105 1.674

VAL_152_vDW 2.1898 0.428 5.114 0.000 1.350 3.029

GLN_36_vDW -0.2015 1.505 -0.134 0.893 -3.151 2.748

LYS_37_vDW -7.6763 1.497 -5.129 0.000 -10.610 -4.742

==============================================================================

Omnibus: 89.437 Durbin-Watson: 1.478

Prob(Omnibus): 0.000 Jarque-Bera (JB): 101.486

Skew: 0.304 Prob(JB): 9.17e-23

Kurtosis: 3.416 Cond. No. 1.43e+16

==============================================================================

**Supplementary Table S2:** Contributions of the residues lining the established FP2's binding pocket subsites towards the favourable binding of E64 to FP2

OLS Regression Results

==============================================================================

Dep. Variable: energy R-squared: 0.453

Model: OLS Adj. R-squared: 0.448

Method: Least Squares F-statistic: 92.18

No. Observations: 4493 AIC: 3.724e+04

Df Residuals: 4452 BIC: 3.750e+04

Df Model: 40 Prob (F-statistic): 0.00

Covariance Type: nonrobust Log-Likelihood: -18579.

===============================================================================

coef std err t P>|t| [95.0% Conf. Int.]

-------------------------------------------------------------------------------

Intercept -18.4067 1.069 -17.226 0.000 -20.502 -16.312

ASN_173 0.1729 0.617 0.280 0.779 -1.036 1.382

ASN_38 -81.7585 15.908 -5.140 0.000 -112.945 -50.572

TRP_43 -0.4607 0.978 -0.471 0.638 -2.378 1.456

GLN_171 10.7413 2.261 4.751 0.000 6.309 15.174

CYS_42 -14.5129 2.792 -5.198 0.000 -19.987 -9.039

ALA_175 -6.7699 1.011 -6.698 0.000 -8.751 -4.788

CYS_39 -4.9479 1.255 -3.944 0.000 -7.408 -2.488

ASP_170 -4.165e-13 9.21e-14 -4.521 0.000 -5.97e-13 -2.36e-13

ASN_87 5.2057 2.814 1.850 0.064 -0.311 10.722

ASN_86 -1.3186 1.101 -1.198 0.231 -3.476 0.839

SER_41 -4.3824 1.073 -4.085 0.000 -6.486 -2.279

THR_233 3.958e-13 7.89e-14 5.014 0.000 2.41e-13 5.51e-13

PHE_75 -1.4794 8.919 -0.166 0.868 -18.966 16.007

LYS_37 -3.2713 7.646 -0.428 0.669 -18.262 11.719

ASN_173_ELE -0.0799 0.022 -3.690 0.000 -0.122 -0.037

ASN_38_ELE 0.0466 0.038 1.229 0.219 -0.028 0.121

TRP_43_ELE -0.2178 0.096 -2.274 0.023 -0.406 -0.030

GLN_171_ELE 0.1107 0.025 4.478 0.000 0.062 0.159

CYS_42_ELE 0.5429 0.064 8.543 0.000 0.418 0.668

ALA_175_ELE -1.8640 0.159 -11.745 0.000 -2.175 -1.553

CYS_39_ELE 0.4420 0.040 11.040 0.000 0.363 0.520

ASP_170_ELE 0.1860 0.017 10.748 0.000 0.152 0.220

ASN_87_ELE 0.3495 0.076 4.599 0.000 0.201 0.499

ASN_86_ELE 0.1030 0.066 1.566 0.117 -0.026 0.232

SER_41_ELE 0.5049 0.100 5.061 0.000 0.309 0.700

THR_233_ELE -0.6508 0.074 -8.761 0.000 -0.796 -0.505

PHE_75_ELE 0.3416 0.100 3.415 0.001 0.145 0.538

LYS_37_ELE -0.7059 0.035 -19.986 0.000 -0.775 -0.637

ASN_173_vDW -0.3436 0.094 -3.672 0.000 -0.527 -0.160

ASN_38_vDW 0.4150 0.229 1.809 0.070 -0.035 0.865

TRP_43_vDW 1.7649 0.171 10.310 0.000 1.429 2.100

GLN_171_vDW 0.4690 0.143 3.276 0.001 0.188 0.750

CYS_42_vDW 0.3745 0.187 2.007 0.045 0.009 0.740

ALA_175_vDW 1.1054 0.261 4.232 0.000 0.593 1.617

CYS_39_vDW -0.4529 0.251 -1.806 0.071 -0.945 0.039

ASP_170_vDW -0.8127 0.466 -1.744 0.081 -1.726 0.101

ASN_87_vDW 3.9788 0.749 5.309 0.000 2.510 5.448

ASN_86_vDW -2.4540 0.447 -5.492 0.000 -3.330 -1.578

SER_41_vDW -0.8867 0.625 -1.418 0.156 -2.112 0.339

THR_233_vDW -0.2469 0.261 -0.946 0.344 -0.758 0.265

PHE_75_vDW 1.9059 0.425 4.482 0.000 1.072 2.740

LYS_37_vDW -8.5593 1.736 -4.929 0.000 -11.964 -5.155

==============================================================================

Omnibus: 16.974 Durbin-Watson: 1.210

Prob(Omnibus): 0.000 Jarque-Bera (JB): 17.045

Skew: 0.149 Prob(JB): 0.000199

Kurtosis: 3.048 Cond. No. 1.48e+16

==============================================================================

**Supplementary Table S3:** Contributions of the residues outside the established FP2's binding pocket subsites towards the favourable binding of E64 to FP2

OLS Regression Results

==============================================================================

Dep. Variable: energy R-squared: 0.598

Model: OLS Adj. R-squared: 0.594

Method: Least Squares F-statistic: 150.2

No. Observations: 4493 AIC: 3.587e+04

Df Residuals: 4448 BIC: 3.616e+04

Df Model: 44 Prob (F-statistic): 0.00

Covariance Type: nonrobust Log-Likelihood: -17889.

===============================================================================

coef std err t P>|t| [95.0% Conf. Int.]

-------------------------------------------------------------------------------

Intercept 5.0263 1.269 3.962 0.000 2.539 7.513

ASP_234 -6.8246 2.003 -3.407 0.001 -10.751 -2.898

LEU_172 -4.2893 0.811 -5.287 0.000 -5.880 -2.699

LEU_84 0.6112 0.377 1.621 0.105 -0.128 1.350

HIS_174 -3.9960 0.526 -7.591 0.000 -5.028 -2.964

GLY_82 -0.9005 0.918 -0.981 0.327 -2.701 0.900

TYR_78 -0.9238 2.633 -0.351 0.726 -6.087 4.239

GLY_40 -6.5219 1.243 -5.248 0.000 -8.958 -4.085

GLY_83 0.5129 1.085 0.473 0.636 -1.614 2.640

TRP_206 29.3879 13.110 2.242 0.025 3.687 55.089

ASN_81 -7.3249 1.781 -4.112 0.000 -10.817 -3.832

CYS_80 -1.104e-13 4.98e-14 -2.217 0.027 -2.08e-13 -1.28e-14

ASN_77 0.1553 3.025 0.051 0.959 -5.775 6.086

SER_149 2.516e-14 8.49e-15 2.965 0.003 8.52e-15 4.18e-14

LYS_76 -7.8156 2.345 -3.333 0.001 -12.412 -3.219

VAL_152 4.178e-14 2.06e-14 2.028 0.043 1.39e-15 8.22e-14

GLN_36 2.002e-13 8.34e-14 2.401 0.016 3.67e-14 3.64e-13

ASP_234_ELE 0.0723 0.007 10.851 0.000 0.059 0.085

LEU_172_ELE 0.3542 0.030 11.870 0.000 0.296 0.413

LEU_84_ELE 1.0770 0.112 9.655 0.000 0.858 1.296

HIS_174_ELE 0.7732 0.046 16.800 0.000 0.683 0.863

GLY_82_ELE 0.3104 0.049 6.370 0.000 0.215 0.406

TYR_78_ELE 0.1756 0.025 7.016 0.000 0.127 0.225

GLY_40_ELE 1.1452 0.043 26.645 0.000 1.061 1.229

GLY_83_ELE 1.1061 0.049 22.728 0.000 1.011 1.202

TRP_206_ELE 0.6983 0.050 14.080 0.000 0.601 0.795

ASN_81_ELE 0.2913 0.024 11.998 0.000 0.244 0.339

CYS_80_ELE 0.2364 0.058 4.080 0.000 0.123 0.350

ASN_77_ELE 0.4392 0.074 5.901 0.000 0.293 0.585

SER_149_ELE -0.0063 0.048 -0.132 0.895 -0.100 0.087

LYS_76_ELE 0.1476 0.022 6.717 0.000 0.105 0.191

VAL_152_ELE 0.3215 0.099 3.245 0.001 0.127 0.516

GLN_36_ELE 0.9235 0.075 12.241 0.000 0.776 1.071

ASP_234_vDW -0.6617 0.093 -7.153 0.000 -0.843 -0.480

LEU_172_vDW 0.8638 0.101 8.537 0.000 0.665 1.062

LEU_84_vDW -0.5616 0.107 -5.232 0.000 -0.772 -0.351

HIS_174_vDW 0.4067 0.109 3.744 0.000 0.194 0.620

GLY_82_vDW 0.1411 0.161 0.875 0.382 -0.175 0.457

TYR_78_vDW 0.7328 0.105 6.955 0.000 0.526 0.939

GLY_40_vDW 1.3117 0.135 9.728 0.000 1.047 1.576

GLY_83_vDW 0.4220 0.111 3.791 0.000 0.204 0.640

TRP_206_vDW 0.0230 0.193 0.119 0.905 -0.356 0.402

ASN_81_vDW -0.1814 0.121 -1.503 0.133 -0.418 0.055

CYS_80_vDW 0.1404 0.299 0.470 0.639 -0.446 0.726

ASN_77_vDW 2.3000 0.374 6.151 0.000 1.567 3.033

SER_149_vDW 0.4156 0.167 2.488 0.013 0.088 0.743

LYS_76_vDW 1.8724 0.469 3.995 0.000 0.954 2.791

VAL_152_vDW 1.0765 0.447 2.410 0.016 0.201 1.952

GLN_36_vDW -0.6335 1.482 -0.428 0.669 -3.538 2.271

==============================================================================

Omnibus: 101.328 Durbin-Watson: 1.330

Prob(Omnibus): 0.000 Jarque-Bera (JB): 145.912

Skew: 0.249 Prob(JB): 2.07e-32

Kurtosis: 3.729 Cond. No. 1.32e+16

==============================================================================


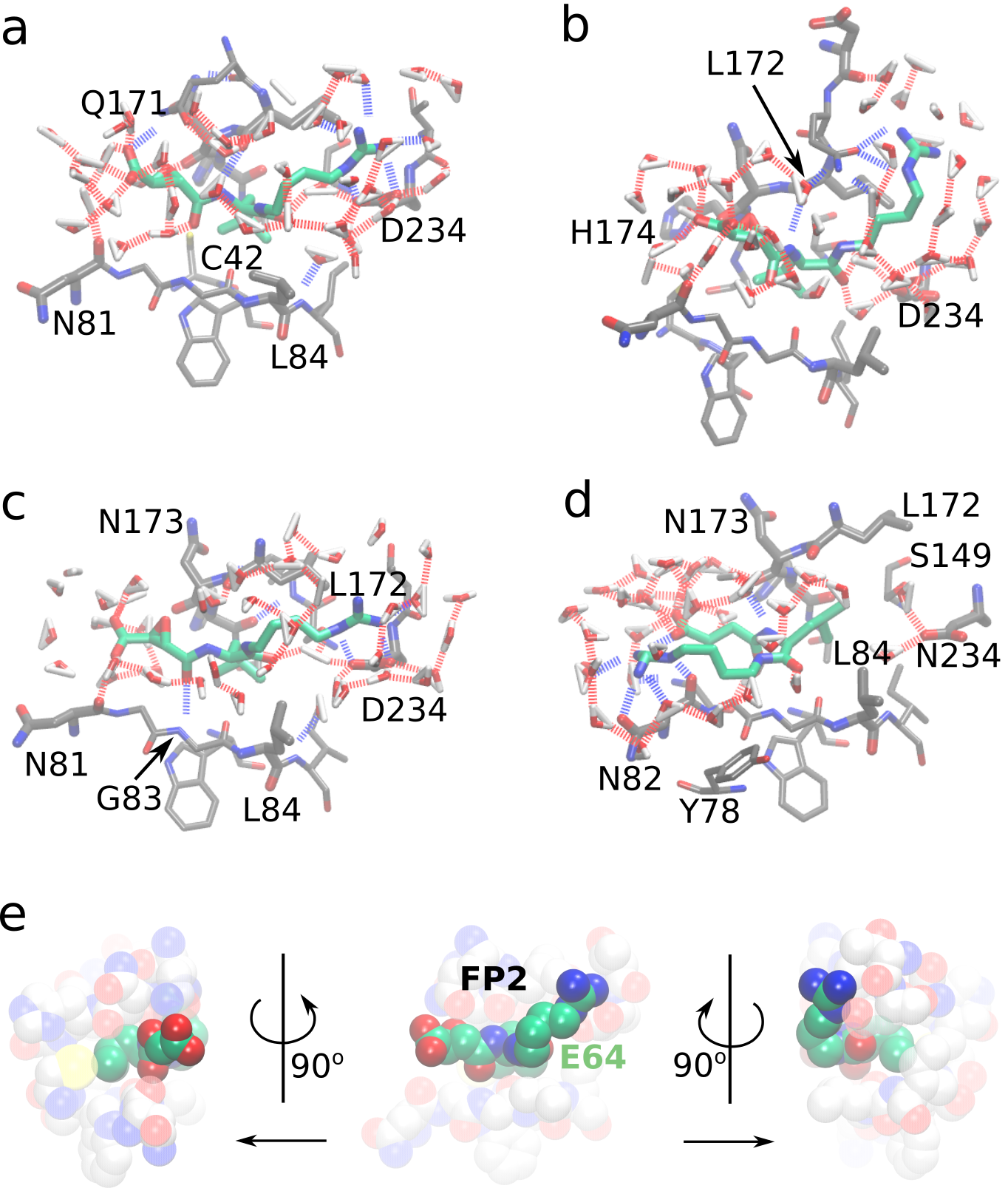


**Supplementary Fig. S1. Hydrogen bonding and other non-bonded interactions between E64 and FP2.** Examples of non-bonded interactions, most especially hydrogen bonding (represented by the broken red and blue lines) and van der Waals interactions between E64 and FP2 are shown here. Panels (a) to (d) show some of the vital roles played by water molecules, wherein water molecules form hydrogen bonds with the residues of FP2 and with some atoms of E64 thereby enhancing the trapping of E64 within the binding cavities of FP2. In each of the cases, we observed favourable van der Waals interactions between E64 and FP2 and noticed that such favourable van der Waals interactions are made possible and enhanced by the flexibility of the backbone of E64 allowing it to flexibly fit into FP2's binding cavity with good surface complementarity as shown in panel (e).

**Supplementary Table S4:** List of residues of FP2 forming hydrogen bonds with E64 through hydrogen bridges created by water molecules

| Residues forming Hydrogen bond (HB) with  E64 through HB bridges built by water | Relative Frequency  (per 100 Frames in the Trajectory) |
| --- | --- |
| N173 | 23.0 |
| N81 | 18.6 |
| L172 | 17.1 |
| D234 | 9.3 |
| D234, S234 ^1^ | 9.0 |
| G83 | 8.2 |
| Y78 | 7.6 |
| Q171 | 6.5 |
| S205 | 5.2 |
| H174 | 5.0 |
| N38 | 4.6 |
| G40 | 2.9 |
| C80 | 2.7 |
| K37 | 2.6 |
| K76 | 2.2 |
| D170 | 2.0 |
| D170, Q205 ^1^ | 1.8 |
| D170, I234 ^1^ | 1.5 |
| S149 | 1.1 |
| S149, K41 ^1^ | 1.1 |
| S149, G43 ^1^ | 1.1 |
| S149, K205 ^1^ | 1.0 |
| C39 | 1.0 |
| N77 | 0.9 |
| S41 | 0.9 |
| S41, Q172 ^1^ | 0.8 |
| W206 | 0.8 |
| N87 | 0.8 |
| V152 | 0.8 |
| G82 | 0.8 |
| G82, H206 ^1^ | 0.7 |
| I85 | 0.7 |
| N86 | 0.5 |
| N86 | 0.5 |

^1^ In some cases, one water molecule forms hydrogen bond bridges between E64 and two residues of FP2.
